# Vacuum Thermoforming Process: An Approach to Modeling and Optimization Using Artificial Neural Networks

**DOI:** 10.3390/polym10020143

**Published:** 2018-02-02

**Authors:** Wanderson de Oliveira Leite, Juan Carlos Campos Rubio, Francisco Mata Cabrera, Angeles Carrasco, Issam Hanafi

**Affiliations:** 1 Departamento de Mecânica, Instituto Federal de Educação, Ciência e Tecnologia de Minas Gerias—Campus Betim, Rua Itaguaçu, No. 595, São Caetano, 32677-780 Betim, Brazil; 2 Escola de Engenharia, Departamento de Engenharia Mecânica, Universidade Federal de Minas Gerais, Av. Pres. Antônio Carlos, No. 6627, Pampulha, 31270-901 Belo Horizonte, Brazil; juan@demec.ufmg.br; 3 Escuela de Ingeniería Minera e Industrial de Almadén, Departamento Mecánica Aplicada e Ingeniería de Proyectos, Universidad de Castilla-La Mancha, Plaza Manuel Meca No. 1, 13400 Ciudad Real, Spain; francisco.mcabrera@uclm.es; 4 Escuela de Ingeniería Minera e Industrial de Almadén, Departamento de Filología Moderna, Universidad de Castilla-La Mancha, Plaza Manuel Meca No. 1, 13400 Ciudad Real, Spain; angeles.carrasco@uclm.es; 5 Ecole Nationale des Sciences Appliquées d’Al Hoceima (ENSAH), Département of Civil and Environmental Engineering, 32000 Al Hoceima, Morocco; hanafi.issam@yahoo.fr

**Keywords:** vacuum thermoforming process, modeling and optimization, artificial neural networks, deviations and process parameters, multi-criteria optimization

## Abstract

In the vacuum thermoforming process, the group effects of the processing parameters, when related to the minimizing of the product deviations set, have conflicting and non-linear values which make their mathematical modelling complex and multi-objective. Therefore, this work developed models of prediction and optimization using artificial neural networks (ANN), having the processing parameters set as the networks’ inputs and the deviations group as the outputs and, furthermore, an objective function of deviation minimization. For the ANN data, samples were produced in experimental tests of a product standard in polystyrene, through a fractional factorial design (2^k-p^). Preliminary computational studies were carried out with various ANN structures and configurations with the test data until reaching satisfactory models and, afterwards, multi-criteria optimization models were developed. The validation tests were developed with the models’ predictions and solutions showed that the estimates for them have prediction errors within the limit of values found in the samples produced. Thus, it was demonstrated that, within certain limits, the ANN models are valid to model the vacuum thermoforming process using multiple parameters for the input and objective, by means of reduced data quantity.

## 1. Introduction

**Figure 1:**
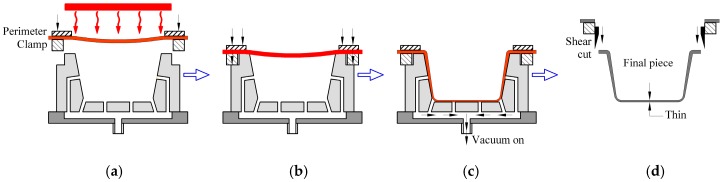
Schematic of basic vacuum thermoforming. (**a**) Heating; (**b**) sealing or pre-stretch; (**c**) forming and cooling; and (**d**) demolding and trimming.

Thermoforming of polymers is a generic term for a group of processes that involves the forming or stretching of a preheated polymer sheet on a mold producing the specific shape. It is considered to be one of the oldest methods of processing plastic materials [1]. The process which uses the vacuum negative pressure force to stretch this heated polymer sheet on a mold is called vacuum forming or vacuum thermoforming [2]. Specifically, this is the forming technique and/or stretching where a sheet of thermoplastic material is preheated by a heating system (Figure 1a,b), and forced against the mold surface (positive or negative) by means of the negative vacuum pressure produced in the space between the mold and sheet (Figure 1c, by mold suction holes and a vacuum pump which “sucks” the air from the space and “pulls” the sheet against the surface of the mold, transferring it, after cooling and removing excess material to shape it (Figure 1d) [3,4]. The typical sequence of this technique by Ghobadnam et al. [5] is presented in Figure 1.

However, what is observed, in practice, is that incorporating prior knowledge or a trial-and-error methods to predict the final result of the process and the quality of the product can be far more difficult. Thus, the evaluation of the final performance of the system is sometimes complex, due to various factors, such as the raw material of the mold, the equipment characteristics, the type and raw material of the sheet, and other factors [6,7,8]. In addition, the process often highlights the conflicts between aspects of quality and adjustments of process control variables [9,10]. In recent years, several authors have developed work with the objective of modelling and predicting the quality of the final product of the vacuum thermoforming process.

Thus, Engelmann and Salmang [6] presented a computational statistics model and data analysis, and Sala et al. [11] and Warby et al. [12] in a complementary focus, worked on the development of an elastic-plastic model for thickness analysis. Many studies concentrated on aspects of mold geometry and process parameters to verify their influence on the wall thickness distribution [5,13,14,15]. A hierarchically-ordered multi-stage optimization strategy for solving complex engineering problems was developed, [3,16]. Martin et al. [17] presented the study of the instrumentation and control of thermoforming equipment and its analysis and control in real-time of multiple variables. The accuracy of the developed controller and its prospective real-time application is evidenced by the results. Some studies focused on modeling, simulation, and optimization of the heating system by different methods and techniques [18,19,20].

However, in complex manufacturing processes such as this, Meziane et al. [21], Tadeusiewicz [22] and Pham [23] suggest that the traditional approaches to process control fail to understand all aspects of process control or existing subsystems. Sometimes the amount and type of variables involved make the computational and mathematical modelling of the system a multi-variate, multi-objective, complex process with non-linear and conflicting objectives [9,10,24]. Thus, according to them, in the last few years, several studies have been presented, using computational intelligence (CI) techniques aimed at the modeling of the non-linear characteristics and conflicting objectives of these processes. The research was carried out using a series of computational tools for the resolution of problems that require human intelligence abilities for their resolution or computational modeling, with artificial neural networks (ANNs) being more intensively investigated and studied [25,26]. 

ANNs are mathematical computational models inspired by biological neural structures or biological neurons [27,28]. The artificial neurons, or perceptron, is constituted of three elements. One input, “*X*”, one weight “*W*”, and a combination of sum function (*φ*) which may be linear or not, and in some cases, a *bias*, *θ_j_*, is included [29]. The “*Y*” response of the ANN is obtained by applying the activation function on the output of the combiner or sum function matrix *Y* = *φ* (*W* × *X* + *θ*) [30]. 

One algorithm model, called a basic ANN, is the multi-layer perceptron (MLP), which is typically composed of combinations of artificial neurons that are interconnected, usually by a node system or mesh. The MLP generally consists of “*n*” neurons interconnected in a system of meshes of nodes and divided into: an input layer, an output layer, and one or more hidden layers, and, between layers, the neurons are connected with their respective weights (biological synapses), which learn or record knowledge (by adjustable weights) between the input and output layers of the network. Furthermore, the network of layers is interconnected externally with their supervised training or learning algorithms [26,27].

In the MLP network, through the input and output data of the network or patterns, the network is trained in a cyclical process by its algorithms and a performance index is calculated for the network in each training round or epoch. These supervised training and learning MLP processes can be continuous until the ANN model “learns” to produce desired outputs for input from its pattern [27] or a performance index of the network, such as the mean square error (MSE), which achieves an error equal to or less than specified, or when the network reaches any other stop criteria specified during model programming. For this, the networks are implemented with training algorithms, the most commonly used being the back propagation (BP) and Levenberg-Marquardt (LM) algorithms. The BP algorithm is a method of supervised learning (batch) that seeks to minimize a global error function or Sum Squared Error (SSE) for the *j* neurons of the layer(s) at each epoch [31,32]. The LM algorithm, developed by Hagan and Menhaj [33] and implemented in MATLAB^®^ software (MathWorks Inc., Natick, MA, USA) by Demuth and Beale [34], is a method that provides a solution to the minimization problem of a non-linear function based on the Gauss-Newton method and gradient descent algorithm via calculation of Jacobian matrices [35]. 

The ability to work with complex or multi-dimensional and multi-criteria problems makes ANNs one of the main methods used in engineering for computational modeling [22]. A model with multi-criteria optimization is defined when it is desired simultaneously to optimize several objective functions and, in some cases, these functions are in conflict, or compete with, each other and, thus, the possible optimal solutions do not allow, for example, the maximization of all the objectives in a joint manner [36].

In this context, some authors have developed computational models based on Computational Intelligence (IC) techniques associated, or otherwise, with statistical optimization for the analysis of quality characteristics of the piece produced by vacuum thermoforming, some of them described by Chang et al. [24]. Likewise, Yang and Hung [9,10] proposed an “inverse” neural network model which was used to predict the optimum processing conditions. The network inputs in this work included the thickness distribution at different positions various parts, and the output or optimal process parameters were obtained by ANNs. Additionally, Küttneret et al. [3] and Martin et al. [17] presented the development of a methodology that uses an ANN to optimize the production technologies together with the product design. Finally, Chang et al. [24] tested an inverse model of ANN on a laboratory scale machine, where it used the desired local thicknesses as inputs and the processing parameters as outputs, with the aim being process optimization.

Thus, first of all, the current work studied both the values of manufacturing parameters and the quality of samples produced by the vacuum thermoforming process on a laboratory scale. Additionally, these initial experimental results were used to investigate the computational modeling of the process through several ANN models that aimed to correctly present the deviation values given a set of manufacturing parameters. These study sequences allowed the study of multivariable and multi-objective optimization algorithms using ANN models to obtain optimum values of the manufacturing parameters simultaneously with the group predictions of product deviations. Finally, validation tests and confirmation are carried out with the objective of evaluating the ability of each model to simulate the process under new experimental conditions and, also, estimate deviations, verify the efficiency of the approach, and validate the proposed methodology.

## 2. Experimental Work

### 2.1. Material, Equipment, and System

For the three-dimensional (3D) design of the model and mold, aspects inherent to the manufacturing process and contraction of 0.5% were considered [8,37] and computer-aided design (CAD) software, integrated with computer-aided manufacturing (CAM), was used. The mold was machined in a computer numeric control (CNC) using plates of medium density fiberboard (MDF) as a raw material. This has dimensional and geometric characteristics of a product standard and, also, a 3D coordinate measuring machine (3D CMM) was used to determine the dimensional and geometric deviations present in the mold. 

A semi-automated vacuum-forming machine was developed and automated by the researchers. This equipment has the capacity to work with plates of thickness of 0.1 to 3.0 mm, a useful area of 280 × 340 mm, a displacement of the mold (*z* axis) of 150 mm, a vacuum pump of 160 mbar with a motor of 1.0 CV, an infrared heating system composed of two resistors of 750 and 1000 W, movement by pneumatic systems, and acquisition of temperature data by “K” thermocouples and non-contact infrared. The system is programmable and controlled by a commercial personal computer (PC) integrated with an Arduino microcontroller (Arduino Company Open Source Hardware, Somerville, MA, USA).

In this work, 2.0 × 2.5 m of white laminated polystyrene (PS) sheets with a thickness of 1.0 mm were used to manufacture the parts. The plates were cut into 300 × 360 (machine size) sheets, cleaned with water and liquid soap of neutral pH, and then dried and packaged in plastic film packages that had previously been heated at 50 °C for two hours.

The commercial equipment and software used in the development of this study are described below and included: a Micro-Hite 3D TESA^TM^ 3D coordinate measuring machine (3D CMM, Hexagon AB, Stockholm, Sweden), Discovery 560 ROMI ^TM^ Machining Center (CNC, INDÚSTRIAS ROMI S.A, São Paulo, Brazil), and Arduino UNO Revision 3 microcontroller board (ATmega328, Arduino Company). A commercial personal computer (PC) environment with Windows^®^ 7 Home Premium 64-bit operating system (Microsoft Company, Redmond, WA, USA), Intel^®^ CoreTM i3-2100 3.10 GHz processor (Intel Corporation, Santa Clara, CA, USA.) and 6 GB of RAM to integrate the machine with the Arduino system’s software and equipment. The software was chosen so that information could be shared, and the main packages used were: Arduino Software (IDE) Release 1.0.5 Revision 2 (Arduino Company) for Arduino microcontroller board, SolidWorks^®^ 2008 (SOLIDWORKS Corp, Waltham, MA, USA), EdgeCAM^®^ 2010 by SolidWorks^®^ (Vero Software, Brockworth, Gloucester, UK), Reflex Software for Micro-Hite 3D TESA^TM^ (Hexagon AB, Stockholm, Sweden), MiniTab 16^®^ (Minitab, Inc., State College, PA, USA), and MATLAB^®^ 2011 version 7. 12. 0. 635 (R2011a) 64-bit (MathWorks Inc.).

### 2.2. Parameters and Measurement Procedure

There is no consensus among authors about the measurement parameters and procedures. According to Küttner et al. [3], Muralisrinivasan [4], Yang and Hung [9,10] and Chang et al. [24] in the vacuum thermoforming process several parameters of control and quality can be used, depending on the type of equipment, mold, and product geometry. Throne [2], Klein [7], Throne [8] and Chang [24] explain that there is no specific measurement procedure or equipment to be used. Thus, they were defined to control the deviations as described in the following paragraphs, with the scales, measurement procedures, and tolerances presented.

For measurement of the errors, 3D MMC was used carrying a 4mm diameter solid probe, calibrated with an error of ±0.004 mm, which has an accuracy of 0.003 mm and CAI software. The reference values for dimensions were calculated, based on the final dimensions of the mold. Additionally, according to Throne [2] and Klein [7], a deviation of ±1% for linear dimension and ±50% for flatness on surfaces are acceptable and, as a reference, the values calculated for dimensions were adopted as the general criteria for acceptance of sample dimensions. 

**Figure 2:**
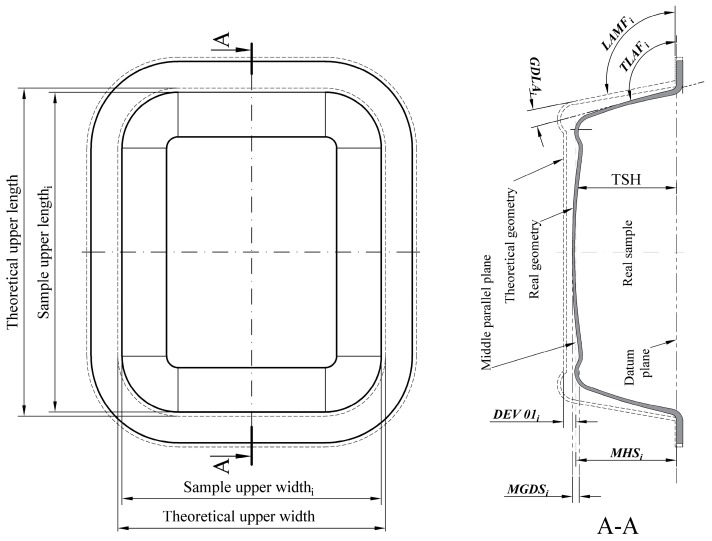
Product standard: dimensions on piece or dimensional deviations parameters.

Figure 2 presents the geometry of the product standard, where dimensions and deviations to be measured in the samples are represented.

The dimensional deviation height (*DDH_i_*) or *DEV 01* was defined as:

(1) $${DDH}_{i}=({MHS}_{i}-TSH)=={DEV01}_{i}=({MHS}_{i}-57.92)$$

where TSH is theoretical sample height and a negative (−) mean value indicates that the height is less than the ideal and a positive mean value (+) that it is greater than the ideal. For the calculation of *DEV 01*, eight (8) points were collected on each surface. Additionally, in all equations in this section, the index *i* represents the *i*-th analyzed sample.

The deviation of the diagonal length (*DDL_i_*) or *DEV 02* is calculated by the difference between the values of the *MLDS_i_* and the value of the *TDL*, being:

(2) $${DDL}_{i}=({MLDS}_{i}-TDL)$$

where *MLDS_i_* is the measured length of the diagonal in the sample, which in this work was defined as the quadratic relation of the lateral distances of the upper end of the sample (length and width) and *TDL* is theoretical diagonal length of the Sample = 207.97mm, so:

(3) $${DDL}_{i}={DEV02}_{i}(\sqrt{{\left({width}_{i}\right)}^{2}+{\left({length}_{i}\right)}^{2}-207.97})$$

For the calculation of *DEV 02*, five points were collected along each lateral of the samples. A negative (−) mean value indicates that the length is smaller than the ideal and a positive mean value that it is greater than the ideal.

The geometric deviation of flatness (*GD_i_*) or *DEV 03*, which will have a zero value (0) for an ideal surface or positive value, was calculated as:

(4) $${GD}_{i}=({MGDS}_{i}-TGDS)=={DEV03}_{i}=({MGDS}_{i}-0.11)$$

where *MGDS_i_* is the measurement geometric deviation flatness in the sample and *TGDS* is the theoretical geometric deviation flatness of the sample, that is, the deviation calculated, which was 0.11 mm. For *DEV 03*, nine (9) points were collected on the lower/bottom surface of the samples.

The *DEV 04* or Geometric Deviation of Side Angles (*GDSA_i_*), in this study, is expressed as:

(5) GDSAi=1z∑J−1ZGDLAi==DEV04i=14∑J−14(LAMFS−TLAFS)

where *z* is the number of sides and *s* the evaluated face. The *GDLA* is the difference between the Lateral Angle Measured on the Face of sample *i* (*LAMF_i_*) and the theoric lateral angle of the face (*TLAF*), for *s* = 1 ... 4, respectively, 95.93°, 95.93°, 96.02°, and 96.06°. For *DEV 04*, nine (9) points were collected on each surface analyzed.

### 2.3. Experimental Study

In this research, we used the manufacturing parameters (factors) described by Throne [2] and compatible with the geometry of sample and equipment, namely: A. heating time (in seconds—*s*); B. electric heating power (in percentage—%); C. mold actuator power (in Bar and cm/s); D. vacuum time (s); E. vacuum pressure (in millibar—mbar). Table 1 shows the levels/values for each parameter.

The experiment was composed of 68 tests according to the planning 2^5-1V^ (fractional factorial design, by Montgomery [38]) with 16 processes of parameter settings and one center point. For each setting and the center point, two (2) replicates were performed in a random sequence. Still, a sample and a repetition were manufactured in the same sequence, totaling 68 pieces (4 samples per processing parameters settings).

The 68 samples of PS were produced and then cooled completely in an air-conditioned room at 22 °C with 60% humidity. After, the inspection methods described in the previous chapter were applied to quantify the linear and geometric dimensions of the samples.

Table 2 shows the types of deviations and respective values of the sample means (by four samplings), the accuracy of this estimate of sample mean (*AE*) and the standard deviation (*S*) of estimate of mean [38], for the 17 process parameters settings tested (center point, test No. 17). It is observed that the data vs. type of deviation are well distributed, except for only one (1) point for *DEV 03*, respectively, standard test 1 (samples 26 and 31 and their repetitions—outlier).

### 2.4. Analysis of Data 

First, the analysis of variance (ANOVA) was developed to test the factors and their effects of first and second order and to evaluate whether each factor was significant or not. The ANOVA results for deviations versus the factors studied are summarized in Table 3, or *F*-test table, with a confidence level of 95% (α = 0.05), and where the critical test value for the *F* distribution is *f*_0,05;1;17_ = 4.45.

In general, for main effects, from Table 3, it can be seen that factors “A” and “B” are the most significant for all deviations and for *DEV 01*. Additionally, for *DEV 02*, the parameter of manufacturing B stands out as significant; for *DEV 03*, all factors are significant; and in *DEV 04*, in sequence, the most significant parameters are B, A, and D. Furthermore, many interaction effects are significant in terms of the deviations. It is concluded that the critical manufacturing parameter for the deviations analyzed are the electric heating power (B) followed by the heating time (A), and also, except for the vacuum pressure factor (E) for the dimensional deviation of the diagonal length (*DEV 02*), at least one factor, or its interaction effect, is significant for one of the deviations.

**Figure 3:**
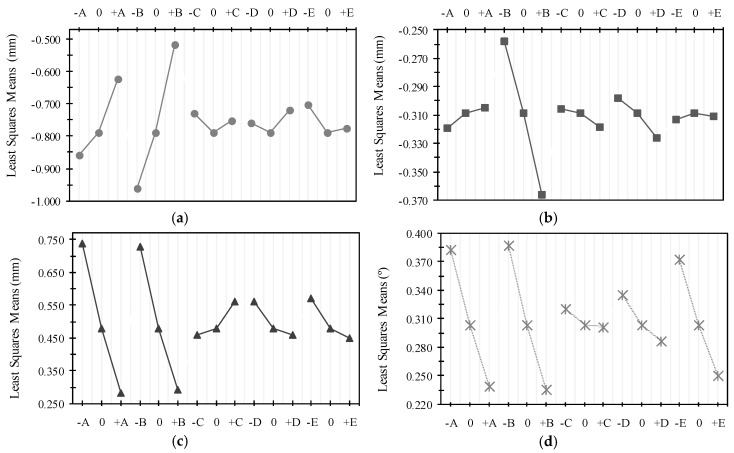
Main experiment: (**a**) *DEV 01* vs. variations of factor levels; (**b**) *DEV 02* vs. variations of factor levels; (**c**) *DEV 03* vs. variations of factor levels; and(**d**) *DEV 04* vs. variations of factor levels.

Figure 3 presents the results of mean deviation values of all factor levels for all factors for each type of deviation. In the figure, we verified that the most relevant factors are those related to heating (A and B). Additionally, in general, it reveals that there is no predominant behavior between factor levels and lower ranges of deviations and the relationships between factors are not proportional. Furthermore, the variation of any input variable (+1 or −1) generates modifications in at least one type of deviation. It can be concluded, in this analysis of data, that the modification of factor levels cannot be studied in isolation for each type of deviation. Therefore, they must be evaluated simultaneously, and also, none of these factors, or their interaction (second-order), can be eliminated from a study or computational modeling of the process since they are significant in at least one type of deviation.

## 3. Development of Modeling and Optimization of Process Based on ANN Models

### 3.1. Modeling, Tests, and Selection of Artificial Neural Network Models

For tests of programming of ANN multilayer models, as input data of the nets, we have used the sequence of factors (process parameters settings) and factor levels of the fractional factorial planning “25^-1V^” with center points, respectively. The output data are the sample means of the results of the deviations (Table 2).

**Figure 4:**
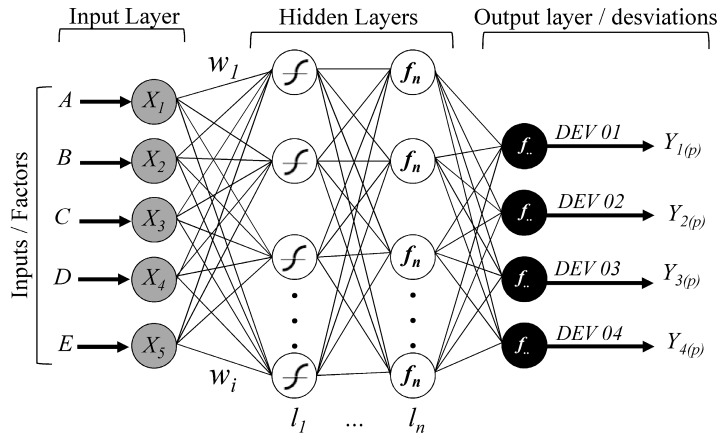
Neural network structure model developed for the tests.

The networks were tested with back propagation and the Levenberg-Marquardt training algorithm. The transfer functions “*tansig*” was used in the first layer and, in the other layers, combinations of the functions “*purelin*” and “*tansig*” were tested. The various network architecture tested were composed of an entrance layer with five data (*Xi*), an exit layer with four values (*Y^l^_j(p_*_)_), and still, *l*-th hidden layer with *j*-th neurons in each. Figure 4 presents the general architecture of the ANN used. 

As general parameters of training of ANNs, the following were used: learning rate = 0.001, ratio to decrease learning rate = 0.001, error maximum increment = 0.001 and network performance = “mae”. As general parameters to stop the network, the following were used: performance goal = 0, minimum performance gradient = 1 × 10^−25^, maximum number of epochs to train = 10000, maximum number of validation increases = 100, and momentum constant maximum = 1 × 10^308^. Additonally, as the mean absolute error (*MAE*) was adopted in substitution of *MSE* as a performance parameter of the network, where *MAE* ≤ 0.145 (General *MAE* of the mean deviation in the samples). Equation (6) describes the calculations of *MAE*.

(6) MAE=1k∑J−1K1n∑i−1n|ej,i|

For the development of multi-criteria optimization algorithms, based on the ANN models, the script codes were implemented and processed using MATLAB^®^ software. In each computational test of a model of optimization, for the patterns shown to the ANN, the four initial solutions and the *MAE* values were recorded. Then a new test of the algorithm was recursively initialized. Where the model reached an improved general value of the *MAE* in a new test run, the code recorded all input and output data of the network and classified it in a sequence of solutions, but, if the *MAE* does not improve, the algorithm continues the tests until it reaches a *net* stop criterion and initializes a new model. At each renewal of the network by a stop criterion, all weights and bias were updated with random values. Each model was tested for even 2000 epochs or for the total time of simulation of 1020 min.

Table 4 summarizes the performance values and processing of main of multi-criteria ANN models and data of the ANNs tested. In this table, we observe the evolution of models by modification of the models’ characteristics, where techniques to improve or simplify the ANN already discussed in other works were applied, along with the change of the training algorithms (model “D”, “K”, etc.), the modification of the *net* structure (model “H”, “M”, etc.), the modification of the transfer function of layers (model “T”, “W”, etc.), the proportional adjustment between the amount the patterns of the network and the number of neuron layers (model “P”, “V”, etc.), and the adjustment of the amount of training data and test data of the models [39,40,41].

**Figure 5:**
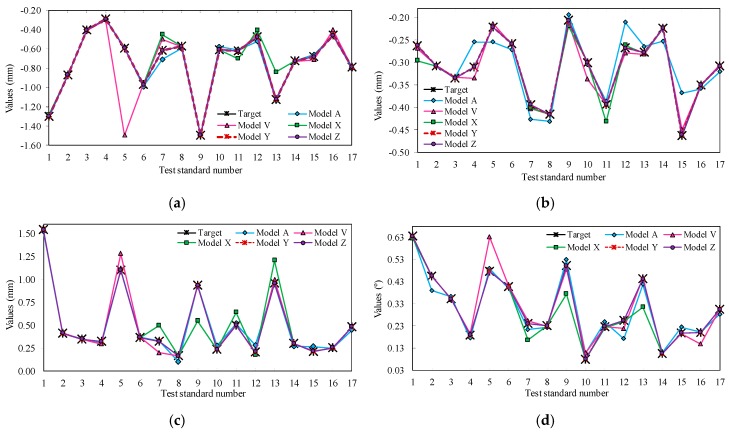
Performance analysis of multi-criteria ANN models—type of deviations vs. predicted values of models vs. target value: (**a**) predicted values of models vs. target values of dimensional deviation height; (**b**) predicted values of models vs. target values of dimensional deviation of the diagonal length; (**c**) predicted values of models vs. target values of geometric deviation of the flatness; (**d**) predicted values of models vs. target values of geometric deviation of the side angles.

In Table 4, the model “A” was the first satisfactory solution (*MAE* ≤ 0.145); however, it presents a *net* structure with many nodes, a considerable number of weights and *bias* and, in addition, a significant amount of processing time, which results in slow computing. The models D, H, K, M, O, P, and T are some intermediate models, but they presented problems that evolved or were improved, such as Model “D” and “K”, that have an *MAE* > 0.145, i.e., with errors of predicted values higher found in the process (Table 2). The V, X, Y, and Z models generally achieved the best performances and predicted values errors considerably lower than the limits found in the process samples. The models are theoretically similar, and present a network structure that simplifies and reduces the processing time, with differences in the training process, the functions used and amount of data. Just as the amount of data and the functions used can modify the models the ANN generated, it cannot be said that the values of the weights and bias are the same, and, consequently, the predicted values (for 68 output data) and the general performance of the ANN*s* are not the same. Figure 5 presents the predicted values by these models and model “A” for each type of deviation and the target values of each pattern. 

As seen in Figure 5, model “A” has significant prediction errors in all deviations, being more evident in *DEV 02* as, for example, data 5 = −0.222 ± 0.010 mm, and in model “A” = −0.254 mm. Model “V” has several errors in the forecasts, highlighting the data value number 5 for *DEV 01* and data value number 5 for *DEV 04*. Of the other models, in general, “X” presents the worst performance in the predictions and one significant prediction error, for test 9 of *DEV 03*, considering the sample variation with value of 0.933° ± 0.132°. Models “Y” and “Z” have negligible errors and, within the ranges found in the samples, are considerably lower when compared with previous values of the other models. The gain in performance value is due to the increase in the number of training data and test data.

**Figure 6:**
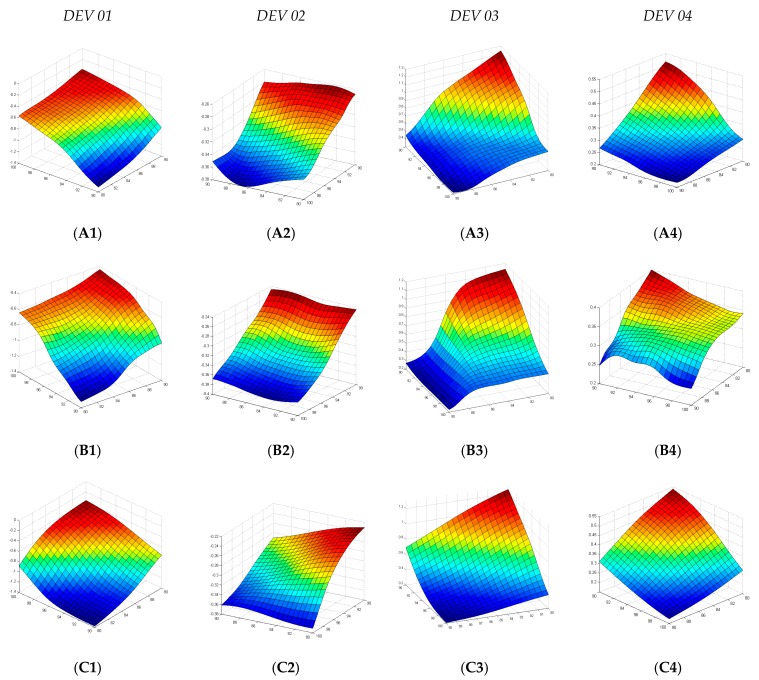
Comparison of the response surfaces of the models for heating time variables vs. electric heating power vs. type of deviations, being: (**A**) “Z” model, (**B**) “Y” model; and (**C**) “V” model, and *DEV 01* is the dimensional deviation height, *DEV 02* is the deviation of diagonal length, *DEV 03* is the geometric deviation of flatness (*GD_i_*), and *DEV 04* is geometric deviation of side angles.

In Figure 6, the response surface of “V”, “Y”, and “Z” models for variables temperature vs. types of deviations are shown. When we compare them, we observed that, although the “V” model has a network structure similar to the “Y” and “Z” models, the use of a linear fit function (*purelin*) in the network contributed to a “linearization” of the surface and the generalization errors (Figure 6(C1–C4)); this was generally observed in other models. Already, the “Y” and “Z” models have hyperbolic tangent sigmoid transfer functions (*tansig*), which contributed to the nonlinear generalization of the models. However, as shown in Figure 6(B1–B4), the amount of data used in model “Y”, up to now, was not adequate to generate an improved model, which was only achieved with the progressive increase of the amount of data of model “Z” (Figure 6(A1–A4)), which makes this model more suitable for this work.

### 3.2. Modeling and Test of Multi-Criteria Optimization Algorithm Models

The multi-criteria optimization algorithms were developed based on the “Z” model (Table 4). The coefficient of performance or the objective function of the algorithm for simultaneous minimization of responses [36] was defined by Equation (7):

(7) $$O_{i}=\frac{1}{8}\sum_{i=1}^{4}\left\{\left(\frac{Y_{i,j(p)}}{admissible\;{erros}_{i}}\right)\;x\;{weights}_{i}\right\}$$

where *j* represents the *j*-th coefficient of performance for a (01) solution vector and *i* the deviation type, where *i* = 1, 2, 3, and 4 for the deviations *DEV 01*, *DEV 02*, *DEV 03*, and *DEV 04*. The values of the “admissible errors” for *i* = 1, 2, …, 4 were defined as |0.6 mm|, |2.1 mm|, |1 mm|, |0.72°|, and the *i*-th weights adopted are: 2, 2, 3, and 1, respectively.

With this data, new codes were programmed with two variations of the algorithm, each with its domain, constraints, and discretization. The data used are described in Table 5.

The two variations of the algorithm were processed according to the same logic, where: the input values for the *j*-th possible solutions were generated in a data matrix, and then the matrix, the ANN model, and the sub-codes were used to find the initial solution. Next, the deviations of this solution were determined and the value of coefficient of performance (*O_j_*) calculated. Finally, the information and data from this possible solution were recorded in a control table in decreasing order. Once this part is processed, the algorithm returns to the first step (internal loop process), repeating the process in search of an improved solution. If it finds one, it writes the data again for this new solution in the decreasing control table. The process was repeated until the model ran in the entire solution space, selected and, thus, found the global minimum value of the solutions vector *O_j_* and the optimal parameters of manufacturing. Table 6 and Table 7 present the best results.

In Table 6 and Table 7 we see that several configurations have the same value of *O_j_*, or very close values, which were already predicted when dealing with a problem with multiple solution spaces, with all being possible optimal solutions to the problem. However, analyzing Figure 3, we see that, in general, for the set of deviations, factor “A” has better results in levels ≥85, factor “B” in levels ≥95, since factor “C” improves next at levels ≤92.5, factor “D” at mean levels ≥8.1, and factor “E” close to levels ≥12.5. From this it follows that the first solution from Table 6 and the sixth solution from Table 7 are the most appropriate solutions to the problem.

### 3.3. Confirmation Experiment

To validate the multi-criteria optimization models developed, new experimental tests were performed, with the respective factors and levels selected. For the processing of the samples, two sequences of tests were performed with the processes of parameter settings or the solutions selected, where five (5) sequentially-manufactured repetitions were performed for each type of setting. Additionally, the same experimental conditions were preserved, as well as the same raw material and infrastructure. In addition, the same steps of the experimental tests were followed. Afterwards, the samples were inspected, adopting the same procedures already described and the deviations previously calculated. 

Table 8 and Table 9 present the results of the expected values of the means of the four deviations for samples in the validation tests, with the 95% confidence interval (CI) on the mean (n = 5 and α = 0.05). The predictions, and the results of the best samples by the *O_j_* value in the main experimental tests, the standard test number being 5, are also shown (Table 2). 

From Table 8 and Table 9 it can be seen that the samples of the validation tests have mean deviations at lower levels than those of the main experimental tests and, also, the CI limit values are at lower levels. This being the case, in relation to the average values, there is a significant improvement of 20% when compared to the best samples of each type test (type A = 18% and Type B = 22.5%). With regard to the predictions of the multi-criteria optimization algorithms models, the deviations predicted by the models are within of CI limits for the validation samples. Additionally, in relation to the means values of these samples the predicted values of the model type “A” have a mean error on average of 13.2% and type “B” o15.5%, both inside the CI. Furthermore, the values of *O_j_* are, on average, 76% below the tolerance limits defined in this work. 

## 4. Conclusions

In general, it is concluded that the work developed with ANN models was able to simultaneously and satisfactorily model the geometric deviations in the polymer vacuum thermoforming process, where there are conflicts of objectives between the quality parameters and the manufacture of the variables using a laboratory infrastructure and with a small number of tests.

The tests allowed us to determine that, to minimize deviations, one should use factor “A” between 85 and 95 s, “B” within the range of 87.5% to 100%, “C” in the range of 85% to 100%, “D” for 6.3 to 8.1, and “E” between 12.5 and 15 mbar. Additionally, the main factors of the analysis of the process are heating time (A) and heating electric power (B). The understanding of their interactions is the critical point for minimizing the set of deviations. In addition, we note that the analysis of results of experimental tests does not allow us to select a (1) single set of factors and levels that simultaneously optimize all parameters. This is because different levels of the same factor could be optimal for different responses, e.g., factor “D” [9].

It has been verified that the gradual modification of the ANN architecture with the modification of functions, algorithms, and the number of layers associated with the progressive increase in the amount of data presented to ANNs significantly reduces the residues and can improve the approximation of the network. Additionally, it can lead to the development of models of optimization by ANNs with reduced numbers of neurons and satisfactory levels of generalization error.

In the validation tests, a gain was obtained in the general minimization of deviations of 20% and coefficient of performance (*O_j_*) of 22.6% and, also, forecast efficiency average values of 84% for the target value. It was verified by CI limit values, that the predicted values by two models are within the expected variability for the process. Additionally, it is concluded that the ANN’s models are an option for the development of algorithms for prediction and optimization of the polymer vacuum thermoforming process with a median amount of data.

Finally, each solution presented by the optimization models represents a (1) set of possible values of the manufacturing parameters within the established modeling criteria, and the choice of one of the solutions will depend on other technical or economic factors involved in the process, such as processing time, operating cost, electric energy consumption, etc.

## Figures and Tables

**Table 1:** Factors and levels selected for the main experiments.

Level	Factors				
	A (s ^a^)	B (% ^a^)	C (bar and cm/s ^a^)	D (s ^a^)	E (mbar ^a^)
1 (−1)	80	90	3.4 and 18.4 (100%)	7.2	10
2 (+1)	90	100	4.0 and 21.6 (85%)	9.0	15

^a^ Unit.

**Table 2:** Experimental main results.

Standard order test	Responses											
	*DEV 01* (mm ^a^)			*DEV 02* (mm ^a^)			*DEV 03* (° ^a^)			*DEV 04* (mm ^a^)		
	Mean ^b^	*AE* ^e^	*S*	Mean ^b^	*AE* ^e^	*S*	Mean ^b^	*AE* ^e^	*S*	Mean ^b^	*AE* ^e^	*S*
1	−1.300	±0.040	0.025	−0.263	±0.039	0.024	1.542 ^c^	±0.104	0.065	0.635	±0.023	0.015
2	−0.871	±0.461	0.290	−0.308	±0.040	0.025	0.411	±0.222	0.139	0.455	±0.098	0.062
3	−0.408	±0.192	0.121	−0.335	±0.253	0.159	0.349	±0.160	0.100	0.351	±0.121	0.076
4	−0.293	±0.327	0.206	−0.310	±0.133	0.084	0.323	±0.134	0.084	0.188	±0.154	0.097
5	−0.596	±0.129	0.081	−0.222	±0.010	0.006	1.100	±0.123	0.077	0.476	±0.066	0.041
6	−0.971	±0.145	0.091	−0.259	±0.035	0.022	0.366	±0.201	0.126	0.407	±0.021	0.013
7	−0.618	±0.131	0.082	−0.395	±0.054	0.034	0.321	±0.470	0.296	0.239	±0.006	0.004
8	−0.576	±0.467	0.293	−0.416	±0.072	0.045	0.164	±0.200	0.125	0.230	±0.020	0.013
9	−1.498	±0.270	0.170	−0.207	±0.087	0.054	0.933	±0.132	0.083	0.501	±0.095	0.060
10	−0.611	±0.283	0.178	−0.301	±0.015	0.010	0.234	±0.152	0.096	0.078	±0.064	0.040
11	−0.625	±0.428	0.269	−0.394	±0.068	0.043	0.500	±0.450	0.283	0.227	±0.007	0.005
12	−0.476	±0.226	0.142	−0.268	±0.038	0.024	0.208	±0.069	0.043	0.253	±0.098	0.061
13	−1.128	±0.241	0.152	−0.278	±0.060	0.038	0.955	±0.364	0.229	0.442	±0.001	0.000
14	−0.728	±0.483	0.303	−0.224	±0.016	0.010	0.297	±0.101	0.063	0.105	±0.067	0.042
15	−0.684	±0.200	0.126	−0.463	±0.028	0.018	0.214	±0.042	0.027	0.198	±0.063	0.039
16	−0.461	±0.449	0.282	−0.350	±0.105	0.066	0.254	±0.031	0.020	0.200	±0.034	0.021
17 ^d^	−0.789	±0.079	0.049	−0.309	±0.019	0.012	0.481	±0.276	0.174	0.304	±0.045	0.029

^a^ Unit; ^b^ Mean average value for four (4) samplings; ^c^ Outlier; ^d^ Center point; ^e^ Accuracy of estimate of sample mean (*AE*) with n = 4 and α = 0.05; *DEV 01*, *DEV 02* and *DEV 04* are in millimeters; *DEV 03* is in decimal degrees.

**Table 3:** ANOVA summary table, results for the deviation analysis vs. factors in main experiments.

Factor	Responses							
	*DEV 01*		*DEV 02*		*DEV 03*		*DEV 04*	
	*F*_(0)_	*p*-Value	*F*_(0)_	*p*-Value	*F*_(0)_	*p*-Value	*F*_(0)_	*p*-Value
A	10.2 ^a^	0.005	0.42	0.542	89.7 ^a^	0.000	77.72 ^a^	0.000
B	37.0 ^a^	0.000	22.5 ^a^	0.000	82.6 ^a^	0.000	86.23 ^a^	0.000
C	0.30	0.592	1.44	0.246	4.6 ^a^	0.046	8.93 ^a^	0.008
D	0.98	0.336	0.02	0.899	6.43 ^a^	0.021	56.03 ^a^	0.000
E	0.08	0.776	0.34	0.567	4.50 ^a^	0.049	1.36	0.259
A*B	1.92	0.184	3.91	0.065	52.1 ^a^	0.000	43.81 ^a^	0.000
A*C	4.86 ^a^	0.042	0.27	0.612	2.73	0.117	6.24 ^a^	0.023
A*D	6.13 ^a^	0.024	2.27	0.150	1.29	0.271	5.58 ^a^	0.030
A*E	1.87	0.189	0.29	0.596	2.63	0.123	2.04	0.171
B*C	5.66 ^a^	0.029	5.04 ^a^	0.038	0.01	0.943	0.42	0.525
B*D	0.05	0.833	0.12	0.739	6.98 ^a^	0.017	30.14 ^a^	0.000
B*E	0.63	0.438	0.89	0.359	0.08	0.783	2.45	0.136
C*D	0.03	0.867	0.14	0.709	1.81	0.196	1.54	0.232
C*E	3.02	0.100	1.12	0.305	2.23	0.154	29.55 ^a^	0.000
D*E	4.89 ^a^	0.041	1.38	0.257	0.37	0.550	0.25	0.817

S = 0.0648608; *R*² = 70.26%; *R*^2^_(*adj*)_ = 42.28% and; ^a^ Significant factors and interaction effect.

**Table 4:** Summary of the main characteristics and performance values of multi-criteria ANN models developed and tested.

Model name	Error model (MAE)	Error model (MSE)	Processing time of Model	No. training data of Model	No. test data of Model	ANN architecture	Network training function of ANN	Transfer function of ANN (1st Layer)	Transfer function of ANN (Layer Hidden)	Best epoch of ANN
Z	0.0001	0.0000001	5.347	14	6	10-8-4	*‘trainlm’*; mu_max = 1 × 10^308^	*‘tansig’*	*‘tansig’*	461
Y	0.0002	0.0000003	6.728	12	4	10-8-4	*‘trainlm’*; mu_max = 1 × 10^308^	*‘tansig’*	*‘tansig’*	873
X	0.0301	0.0000163	8.004	11	3	10-8-4	‘*‘trainlm’*; mu_max = 1 × 10^308^	*‘tansig’*	*‘tansig’*	832
W	0.0877	0.0720541	39.575	11	3	10-8-4	*‘traingd’*; *η* = 0.001; *ρ* = 0.001; *τ* = 0.001;	*‘tansig’*	*‘tansig’*	10359
V	0.0303	0.0000795	6.192	11	3	10-8-4	*‘trainlm’*; mu_max = 1 × 10^308^	*‘tansig’*	*‘purelin’*, *’tansig’*	685
T	0.0164	0.0000976	220.040	11	3	16-8-4	*‘trainlm’*; mu_max = 1 × 10^308^	*‘tansig’*	*‘purelin’*, *’tansig’*	19855
P	0.0319	0.0000000	58.800	11	3	5-4-8-4	*‘trainlm’*; mu_max = 1 × 10^308^	*‘tansig’*	*‘purelin’, ‘tansig’, ’purelin’*	762
O	0.0085	0.0000105	64.461	11	3	8-8-8-4	*‘trainlm’*; mu_max = 1 × 10^308^	*‘tansig’*	*‘purelin’*, *‘tansig’*, *’purelin’*	4482
M	0.0320	0.0000620	140.268	11	3	16-8-8-4	*‘trainlm’*; mu_max = 1 × 10^308^	*‘tansig’*	*‘purelin’*, *‘tansig’*, *’purelin’*	7444
K	0.1529	0.1669912	74.772	11	3	24-12-8-4	*‘traingd’*; *η* = 0.001; *ρ* = 0.001; *τ* = 0.001;	*‘tansig’*	*‘purelin’*, *‘tansig’*, *’purelin’*	11882
H	0.0256	0.0000000	490.485	11	3	24-12-8-4	*‘trainlm’*; mu_max = 1 × 10^308^	*‘tansig’*	*‘purelin’*, *‘tansig’*, *’purelin’*	9340
D	0.1832	0.1938314	7.900	11	3	32-16-8-4	*‘traingd’*; *η* = 0.001; *ρ* = 0.001; *τ* = 0.001;	*‘tansig’*	*‘purelin’*, *‘tansig’*, *’purelin’*	1656
A	0.02135	0.0005825	205.544	11	3	32-16-8-4	*‘trainlm’*; mu_max = 1 × 10^308^	*‘tansig’*	*‘purelin’*, *‘tansig’*, *’purelin’*	3507

**Table 5:** Restrictions domain used for optimization model “A” and model “B”.

Optimization model	Factor	Constraints			Generated points
		Domain		Discretization	
		≤ X_i_ ≤		Unit	
Variation“A”	A	80	90	5	3
	B	90	100	5	3
	C	85	100	7.5	3
	D	7.2	9.0	0.9	3
	E	10	15	2.5	3
				Total	243
Variation“B”	A	75	95	2.2	10
	B	85	105	2.5	9
	C	77.5	100	2.5	10
	D	6.3	9.9	0.9	5
	E	7.5	15	1.25	7
				Total	31500

**Table 6:** Summary of the 10 best results of the “A” variation of the optimization algorithm.

Solution	Factor					*O*_*j*(*p*)_
	*A* (*s*)	*B* (%)	*C* (%)	*D* (*s*)	*E* (*mbar*)	
1st	90	100	100	8.1	12.5	0.27
2nd	90	100	92.5	7.2	12.5	0.27
3rd	85	100	100	7.2	12.5	0.27
4th	90	95	100	8.1	12.5	0.28
5th	90	100	85	8.1	10	0.28
6th	90	95	100	7.2	12.5	0.28
7th	85	95	100	7.2	12.5	0.28
8th	90	95	92.5	7.2	12.5	0.29
9th	90	100	100	7.2	12.5	0.29
10th	85	95	100	7.2	12.5	0.30

**Table 7:** Summary of the 10 best results of the “B” variation of the optimization algorithm.

Solution	Factor					*O*_*j*(*p*)_
	*A* (*s*)	*B* (%)	*C* (%)	*D* (*s*)	*E* (*mbar*)	
1st	92.6	90	100	7.2	12.5	0.24
2nd	95	90	100	8.1	12.5	0.24
3rd	95	87.5	100	7.2	12.5	0.24
4th	95	90	100	7.2	12.5	0.24
5th	95	87.5	100	6.3	10	0.24
6th	95	90	96.25	8.1	12.5	0.24
7th	95	87.5	96.25	6.3	10	0.24
8th	92.6	90	96.25	7.2	12.5	0.24
9th	92.6	87.5	100	7.2	12.5	0.24
10th	95	87.5	100	8.1	12.5	0.24

**Table 8:** Comparative results of the multi-criteria optimization model type “A”.

	Validation samples ^a^			Model type “A”	Main experimental n° 04 ^b^		
	Mean	95% CI		Predicted	Mean	95% CI	
***DEV 01***	−0.255	−0.298	−0.213	−0.294	−0.293	−0.620	0.034
***DEV 02***	−0.341	−0.419	−0.263	−0.376	−0.310	−0.444	−0.177
***DEV 03***	0.193	0.156	0.231	0.185	0.323	0.189	0.456
***DEV 04***	0.134	0.050	0.218	0.188	0.188	0.034	0.342
***O_j_***	0.23	0.17	0.30	0.27	0.31	0.39	0.27

^a^ For validation samples n = 5 and α = 0.05; ^b^ For the main experiment n = 4 and α = 0.05; *DEV 01*, *DEV 02* and *DEV 04* are in millimeters; *DEV 03* is in decimal degrees.

**Table 9:** Comparative results of the multi-criteria optimization model type “B”.

	Validation Samples ^a^			Model Type “B”	Main Experimental n° 04 ^b^		
	Mean	95% CI		Predicted	Mean	95% CI	
***DEV 01***	−0.366	−0.480	−0.252	−0.293	−0.293	−0.620	0.034
***DEV 02***	−0.246	−0.267	−0.225	−0.242	−0.310	−0.444	−0.177
***DEV 03***	0.108	0.078	0.139	0.182	0.323	0.189	0.456
***DEV 04***	0.136	0.068	0.204	0.099	0.188	0.034	0.342
***O_j_***	0.25	0.17	0.33	0.24	0.31	0.39	0.27

^a^ For validation samples n = 5 and α = 0.05; ^b^ For the main experiment n = 4 and α = 0.05; *DEV 01*, *DEV 02* and *DEV 04* are in millimeters; *DEV 03* is in decimal degrees.
